# FXR, a Key Regulator of Lipid Metabolism, Is Inhibited by ER Stress-Mediated Activation of JNK and p38 MAPK in Large Yellow Croakers (*Larimichthys crocea*) Fed High Fat Diets

**DOI:** 10.3390/nu13124343

**Published:** 2021-12-01

**Authors:** Jianlong Du, Xiaojun Xiang, Dan Xu, Junzhi Zhang, Wei Fang, Wei Xu, Kangsen Mai, Qinghui Ai

**Affiliations:** 1 Key Laboratory of Aquaculture Nutrition and Feed (Ministry of Agriculture and Rural Affairs), Key Laboratory of Mariculture (Ministry of Education), Ocean University of China, 5 Yushan Road, Qingdao 266003, China; dujianlong@ouc.edu.cn (J.D.); xxj_ouc@163.com (X.X.); danxu_ouc@163.com (D.X.); zhjun123@hunau.edu.cn (J.Z.); fw17852329921@163.com (W.F.); weixu@ouc.edu.cn (W.X.); kmai@ouc.edu.cn (K.M.); 2 Laboratory for Marine Fisheries Science and Food Production Processes, Qingdao National Laboratory for Marine Science and Technology, 1 Wenhai Road, Qingdao 266237, China

**Keywords:** high-fat diets, FXR, lipid metabolism, ER stress, MAPK

## Abstract

High-fat diets induced abnormal lipid accumulation in the liver of cultured fish that caused body damage and diseases. The purpose of this research was to investigate the role and mechanism of farnesoid X receptor (FXR) in regulating lipid metabolism and to determine how high-fat diets affect FXR expression in large yellow croakers. The results showed that ligand-meditated FXR-activation could prevent abnormal lipid accumulation in the liver and hepatocytes of large yellow croakers. FXR activation increased the expression of lipid catabolism-related genes while decreasing the expression of lipogenesis-related genes. Further investigation found that the promoter activity of proliferator-activated receptor α (PPARα) could be increased by croaker FXR. Through the influence of SHP on LXR, FXR indirectly decreased the promoter activity of sterol regulatory element binding protein 1 (SREBP1) in large yellow croakers. Furthermore, the findings revealed that endoplasmic reticulum (ER)-stress-induced-activation of JNK and P38 MAPK participated in the reduction of FXR induced by high-fat diets. Then, hepatocyte nuclear factor 1α (HNF1α) was confirmed to be an FXR regulator in large yellow croaker, and it was reduced by high-fat diets and ER stress. In addition, co-expression of c-Jun with HNF1α inhibited the effect of HNF1α on FXR promoter, and suppression of P38 MAPK could relieve the HNF1α expression reduction caused by ER stress activation. In summary, the present study showed that FXR mediated lipid metabolism can prevent abnormal lipid accumulation through regulating PPARα and SREBP1 in large yellow croakers, while high-fat diets can suppress FXR expression by ER stress mediated-activation of JNK and P38 MAPK pathways. This research could benefit the study of FXR functions in vertebrate evolution and the development of therapy or preventative methods for nutrition-related disorders.

## 1. Introduction

Recently, high-fat diets (HFDs) have become increasingly common in aquaculture. However, excessive fat intake leads to abnormal lipid deposition in cultured fish liver, which may cause body damage, diseases and result in retarded growth. In mammals, the issue of excessive lipid accumulation in the liver is also present in nonalcoholic fatty liver disease (NALFD) [1]. On the basis of googd understanding of the functions of metabolic genes, various metabolic targets have been studied and used for regulating lipid metabolism and treatment of NALFD [2]. However, the understanding of the mechanism of lipid metabolism and the roles of genes in fish is still limited. Thus, there is an urgent need to investigate the metabolic process and develop solutions for managing lipid deposition in fish.

Farnesoid X receptor (NR1H4) is considered to be an essential regulator of lipid metabolism in animals [3,4]. FXR knockout (KO) mice display hepatosteatosis and hypertriglyceridemia [5,6]. Ligand-mediated FXR activation, in obese mice, has also been shown to reduce triglyceride accumulation and prevent liver steatosis [5,7,8,9,10]. FXR is a transcription factor that is activated by ligands and regulates the expression of downstream genes [4]. Currently, it has been developed as an important metabolic target to treat lipid metabolism-related diseases [11]. However, little is known about fish FXR protein. Limited studies have shown the discrepancies in the tissue distribution and ligand selectivity of FXR that exist among different fish in evolution. FXR is abundant in the liver and intestines of medaka, skates, and large yellow croaker, but not in the liver of pufferfish [12,13,14,15]. In addition, FXR of skates did not respond to the natural ligands (chenodeoxycholic acid, and cholic acid) and synthetic agonist (GW4064) in mammals [15], while FXR in pufferfish, medaka, large yellow croaker and yellow catfish can be activated by chenodeoxycholic acid (CDCA) or GW4064 [16,17,18,19,20]. Previous research has found that FXR expression is reduced in NAFLD, mice and fish fed high-fat diets [21,22,23], but there has been little research that explores how high-fat diets affect FXR expression. A study in aging mice reported that activation of endoplasmic reticulum (ER) stress contributes to a decrease in hepatic FXR expression [24]. In addition, high-fat diets have been shown to induce ER stress in the liver of mice and fish [25,26,27]. However, the role of FXR in regulating lipid metabolism in fish and whether high-fat diets decrease the expression of FXR via ER stress remains unknown.

The large yellow croaker (*Larimichthys crocea*) is a commercially important marine fish that is widely cultured in China. Abnormal lipid accumulation in the liver is common in large yellow croakers. Methods for large yellow croaker cell culture are well developed [28]. As a result, large yellow croaker is a good model for understanding the mechanisms and regulation of FXR in lipid metabolism. Therefore, the purpose of this study was to see if FXR could be used as a therapeutic target to regulate lipid metabolism and how HFD feeding decreases the expression of FXR in fish.

## 2. Method

### 2.1. Animal Feeding Experiment

The Laboratory Animal Management Rule (Chinese Order No. 676 of the State Council, revised 1 March 2017) was followed during the experiment. Four diets were created as follows: a control diet (Control, 12% lipid), a high-fat diet (HFD, 18% lipid), and an HFD diet supplemented with 300 or 900 mg CDCA/kg (HFD-CDCA300 or HFD-CDCA900) (Supplementary Table S1). Xiangshan Harbor Nursery Co., Ltd. provided the same batch of juvenile yellow croakers (Ningbo, China). The fish (initial weight: 10.03 ± 0.02 g, 4 months) were fed commercial feed for two weeks prior to the experiment. Fish were distributed at random to 12 floating cages (1 × 1 × 1.5 m, 60 fish per cage), with each diet being assigned to cages in triplicate. The experiment was carried out under appropriate conditions [29] for a period of ten weeks. After the feeding experiment, the fish were fasted for 24 h before being anesthetized with eugenol (1:10,000). Then, the muscle and liver tissues (6 individuals per cage) were then collected and frozen in liquid nitrogen before being stored at −80 °C in a freezer.

### 2.2. Cell Culture and Treatment

Large yellow croaker liver cell line (LYCL) cells were cultured in DMEM/F12 supplemented with 15% fetal bovine serum (FBS) (Biological Industries, Israel), 100 U/mL penicillin and 100 μg/mL streptomycin at 27 °C and 5% CO_2_. The HEK 293T cells and primary hepatocytes from large yellow croakers were cultured, following previously published methods [28,30].

To find out how FXR affected lipid metabolism, LYCL cells were injected with recombinant adenovirus encoding croakers FXR (advFXR) for 36 h, and then stimulated with fatty acids (FA) (palmitic acid (PA)/oleic acid = 1:1, 200 μM) for 24 h. Primary hepatocytes were treated with CDCA (0, 50, 100, 150 and 200 μM) or GW4064 (0, 0.5, 1, 2, and 4 μM) for 24 h. In the LYCL cells and primary hepatocytes, the short interfering RNA targeting fxr [28] was transfected for 36 h using a Lipofec-tamine^®^RNAiMAX transfection reagent (Invitrogen, Carlsbad, CA, USA) to knock down fxr mRNA expression. The LYCL cells were treated for 24 h with palmitic acid (PA, 100 μM, Sigma, St. Louis, MO, USA), thapsigargin (TG, 1 μM, MCE, Bloomfield, NJ, USA), tunicamycin (TM, 4 μM, MCE, Bloomfield, NJ, USA) and tauroursodeoxycholate (TUDCA, 0, 50, 100, 150 and 200 μM, MCE, Bloomfield, NJ, USA) to study the effect of ER stress on *fxr* expression. To confirm the change and role of the MAPK pathway in ER-stress-regulated expression of *fxr*, the cells were treated with PA, TG, or TM for 6 h for SP600125 (JNK inhibitor, 5 μM, Sigma, St. Louis, MO, USA), SB23580 (P38 inhibitors, 5 μM, Sigma, St. Louis, MO, USA), and PD98059 (ERK inhibitors, 5 μM, Sigma, St. Louis, MO, USA) for 1 h, and then stimulated with TG for 6 h.

### 2.3. Quantitative Real-Time PCR

Gene expression was examined as described by Du et al. [29]. To identify the expression of lipid metabolism and ER stress-related genes, specific primers were created and listed in Supplementary Table S2. The β-actin and *GAPDH* genes were utilized as housekeeping genes. The 2^−ΔΔCT^ method was used to determine and normalize the gene expression levels.

### 2.4. Plasmid Construction and Luciferase Reporter Assay

The promoter of croaker PPARα, ATGL and SREBP1 were cloned into the reporter plasmid (pGL3-basic vector) by a ClonExpress II One Step Cloning Kit (Vazyme, Nanjing, China) (Supplementary Table S3). Expression plasmids (pCS2-FXR, pCS2-FXR-FLAG, pCS2-SHP, pCS2--SHP-GFP, pCS2-LXRα, pCS2-LXRα-HA, pCS2-PPARα and pCS2-HNF1α) and reporter plasmids of promoter (pGL3-SCD1 and pGL3-FAS) were stored in our laboratory. DNA sequencing was used to confirm all plasmids, and an EasyPure HiPure Plas-mid MiniPrep kit was used to manufacture them (TransGen Biotech, Beijing, China).

The HEK 293T cells were co-transfected with reporter plasmids, phRL-CMV plasmid, and expression plasmid to examine the impact of expression plasmids on the promoter activity of report plasmids. A TransDetect double-luciferase reporter assay kit (TransGen Biotech, Beijing, China) was used to assess luciferase activity.

### 2.5. Western Blotting

The protein level was determined, according to the method of Tan et al. [31]. The antibodies used in this study included: PPARα (Absin, Shanghai, China), CPT1 (Proteintech, USA), FXR (Bioss, Beijing, China), HNF1α (Abcam, Cambridge, UK), GRP78 (Cell Signaling Technology, CST, Danvers, MA, USA), p-PERK (Bioss, Beijing, China), p-EIF2α (CST, Danvers, MA, USA), JNK (CST, Danvers, MA, USA), p-JNK (CST, Danvers, MA, USA), P38 (CST, Danvers, MA, USA), p-P38 (CST, Danvers, MA, USA), ERK(CST, Danvers, MA, USA), p-ERK(CST, Danvers, MA, USA), c-Jun (CST, Danvers, MA, USA), p-c-Jun (CST, Danvers, MA, USA), GAPDH (ZSGB-Bio, Beijing, China), FLAG (CST, Danvers, MA, USA), HA (CST, Danvers, MA, USA) and GFP (CST, Danvers, MA, USA). An electrochemiluminescence kit (Beyotime, Beijing, China) was used to visualize the immunoreactive protein, which was then scanned with an Epson Perfection V33 scanner.

### 2.6. Chromatin Immunoprecipitation Assay

The HEK 293T cells were co-transfected with pGL3-PPARα promoter plasmid and pCS2-FXR-FLAG plasmid to determine the binding effect of FXR on the promoter of PPARα in large yellow croaker. After 48 h of transfection, the HEK293 cells were fixed with 1% formaldehyde at 37 °C for 10 min. Then, a chromatin immunoprecipitation assay (ChIP) was performed, according to the ChIP Kit (Thermo Fisher Scientific, Waltham, MA, USA) protocol.

### 2.7. Immunoprecipitation

The immunoprecipitation experiments were performed as described by Du et al. [20]. In brief, cells were co-transfected with LXRα-HA and SHP-GFP plasmids for 48 h. Then, cells were washed and collected. The supernatant was treated overnight at 4 °C with IgG-beads (Sigma, Missouri, USA), GFP antibody (CST, Danvers, MA, USA) and Pierce^®^ANTI-HA agarose (Thermo, Waltham, MA, USA). Then, the fusion protein was eluted for immunoblotting after washing.

### 2.8. Statistical Analysis

SPSS 20.0 was used to analyze the data, which are presented as the means ± SEMs. Statistical differences among groups were determined by one-way ANOVA, followed by Duncan’s multiple range test, with *p* set at <0.05. Student’s *t*-tests were used to examine the significance of differences between groups.

## 3. Result

### 3.1. CDCA Supplementation Decreases HFD-Induced Lipid Deposition in the Liver

**Figure 1:**
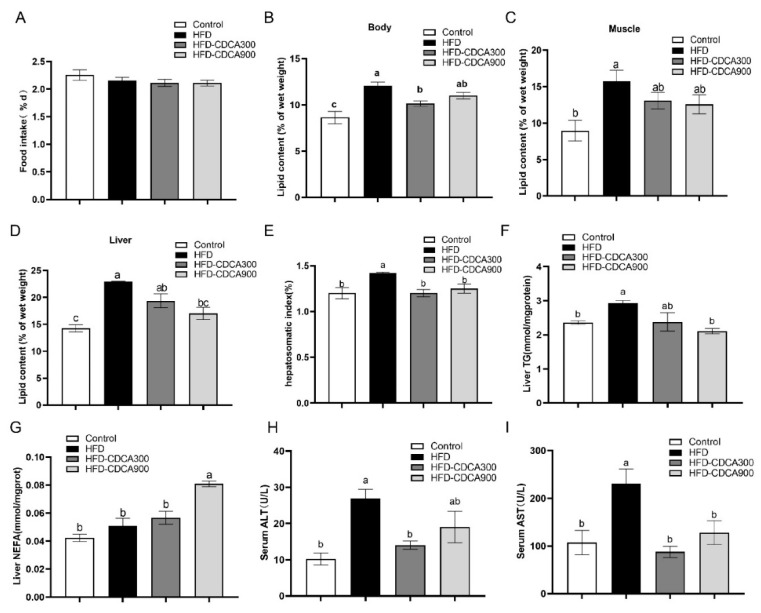
Effects of HFD and supplementation of CDCA on: (**A**) Food intake; (**B**–**D**) lipid content of whole body, muscle, and liver; (**E**) hepatosomatic index; (**F**,**G**) TG and NEFA of liver; (**H**,**I**) ALT and AST of serum in large yellow croakers. The data are provided as means ± SEMs (*n* = 3) and were analyzed using Duncan’s multiple range test. The letters a and b indicate that there is a significant difference between means, *p* < 0.05.

The in vivo results showed that HFD and CDCA supplementation had no significant effects on the survival, weight gain rate (Supplementary Figure S1), and food intake (*p* < 0.05) (Figure 1A). HFD significantly increased the lipid content of the whole body, muscle, and liver, as well as the hepatosomatic index and liver TG content as compared with the control group (*p* < 0.05) (Figure 1B–F). The HFD group had significantly higher serum ALT and AST levels than the control group (*p* < 0.05) (Figure 1H,I), which indicated that high-fat diets may cause liver injury. In addition, as compared with the HFD group, CDCA supplementation (HFD-CDCA 300 or HFD-CDCA 900) significantly reduced the lipid content of whole body and liver, hepatosomatic index, liver TG content, and serum ALT and AST, and increased the NEFA content of liver (*p* < 0.05) (Figure 1B–I). These findings suggest that FXR activation in vivo could improve HFD-induced liver lipid deposition and liver injury.

### 3.2. Effects of HFD and CDCA Supplementation on Lipid Metabolism-Related Gene Expression in the Liver

**Figure 2:**
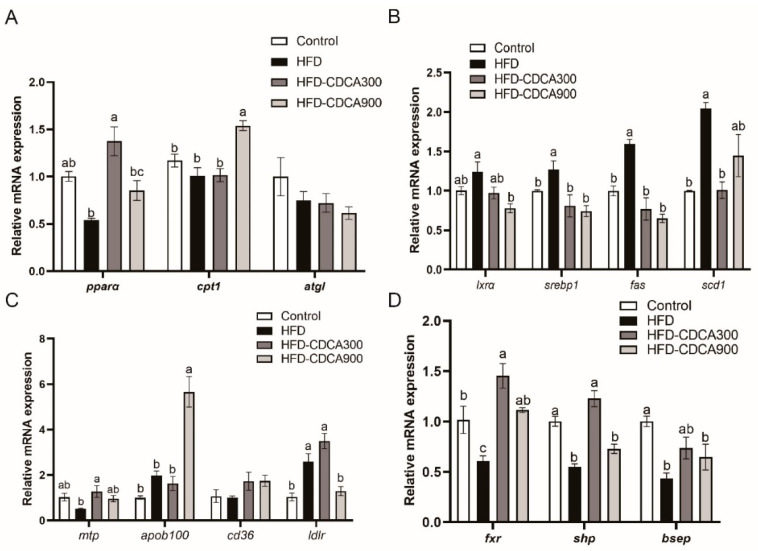
Effect of HFD and CDCA supplementation on expression of lipid metabolism related genes. Expression of genes involved in: (**A**) Lipolysis and β-oxidation; (**B**) lipogenesis; (**C**) transport. (**D**) Expression of *fxr* and target genes (*shp* and *bsep*). The data are provided as means ± SEMs (*n* = 3) and were analyzed using Duncan’s multiple range test. The letters a–c indicate that there is a significant difference between means, *p* < 0.05.

To explore the mechanism of FXR activation on hepatic lipid metabolism, the expression of related genes was detected. The results showed that high-fat diets significantly enhanced the mRNA level of lipogenesis related gene (*srebp1*, *fas* and *scd1)* (*p* < 0.05) (Figure 2B). Although not statistically significant, the HFD group had lower expression of *pparα* and *mtp*, two essential regulated genes involved in β-oxidation and transport, respectively, than the control group (*p* > 0.05) (Figure 2A,C). CDCA supplementation significantly increased expression of genes involved in β-oxidation (*pparα* and *cpt1*), transport (*mtp*, *apob100* and *ldlr*), and decreased expression of lipogenesis-related genes (*lxrα*, *srebp1*, *fas* and *scd1*) as compared with the HFD group (*p* < 0.05) (Figure 2A–C). Furthermore, mRNA expression of fxr and FXR target genes (*shp* and *bsep*) was significantly lower in the HFD group than the control group, but significantly upregulated upon CDCA supplementation (*p* < 0.05) (Figure 2D).

### 3.3. Overexpression and Agonists-Meditated Activation of FXR Decreases Triglycerides Concentration In Vitro

**Figure 3:**
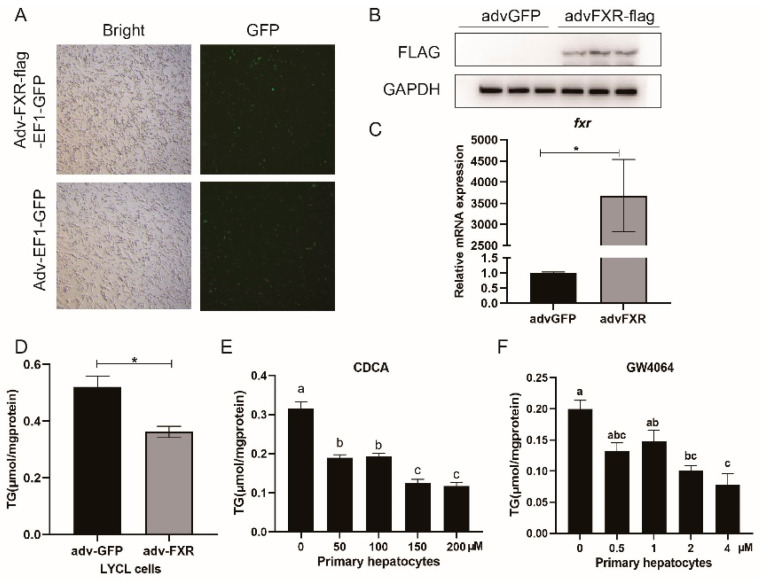
Effects of FXR overexpression or ligand-mediated FXR activation on the TG content of LYCL cells and primary hepatocytes of large yellow croakers: (**A**–**C**) Fluorescence analysis, immunoblots, and qRT-PCR assays for FXR in LYCL cells infected by croaker *fxr* adenovirus; (**D**–**F**) TG content of LYCL cells with overexpression of FXR or after treatment with CDCA and GW4064. Data are shown as means ± SEMs (*n* = 3) and were analyzed using Duncan’s multiple range test and Student’s *t*-test. The letters a–c indicate that there is a significant difference between means, * *p* < 0.05.

To overexpress the croaker FXR gene, the LYCL cells were infected with recombinant adenovirus, and primary hepatocytes were treated with CDCA or GW4064. The results showed that adenovirus infection significantly increased FXR protein and mRNA levels in LYCL cells (*p* < 0.05) (Figure 3A,B). FXR overexpression reduced triglyceride content in LYCL cells, while in primary hepatocytes, CDCA or GW4064 treatment also could reduce the triglyceride content (*p* < 0.05) (Figure 3D–F).

### 3.4. Regulation of FXR on Expression of pparα and Other Lipid Catabolism-Related Genes

**Figure 4:**
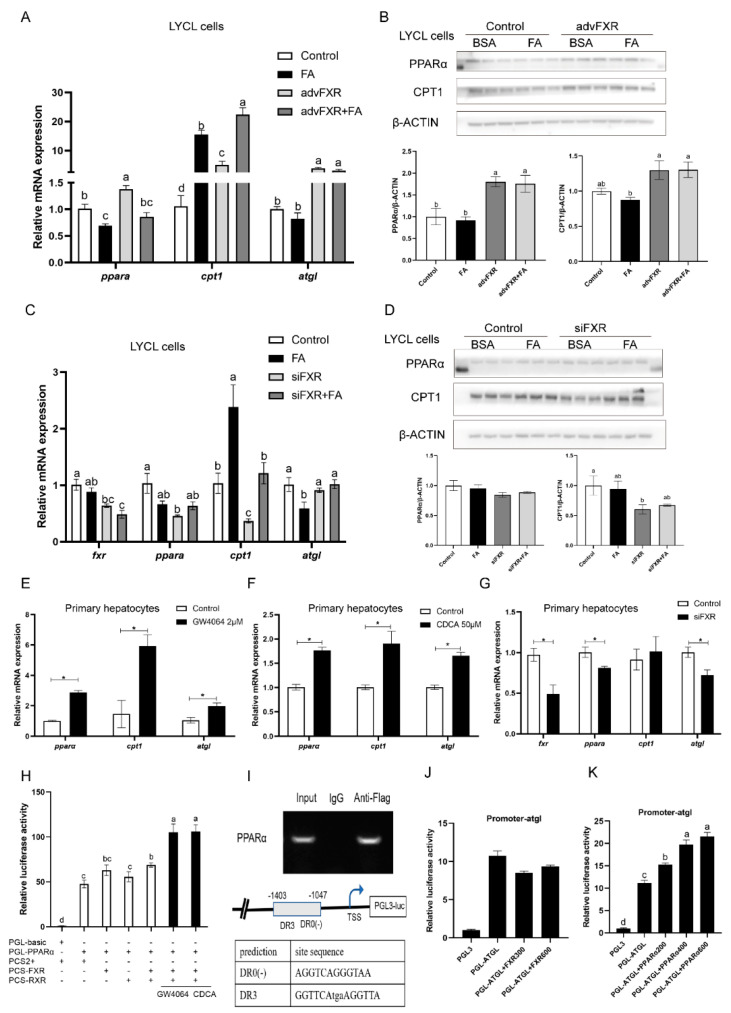
FXR affects genes involved in lipolysis and β-oxidation of large yellow croakers: (**A**,**B**) qRT-PCR assays or immunoblots for PPARα, CPT1, and ATGL in LYCL cells after FXR overexpression and treatment with FA; (**C**,**D**) qRT-PCR assays or immunoblots for PPARα, CPT1, and ATGL in LYCL cells after FXR knockdown and treatment with FA; (**E**–**G**) gene expression of *pparα*, *cpt1*, and *atgl* in primary hepatocytes after GW4064 or CDFA treatment or FXR knockdown; (**H**) promoter activity of croaker PPARα in HEK 293T cells after FXR overexpression and treatment with FXR agonists; (**I**) ChIP analysis and identification of the FXR binding site in the croaker PPARα promoter region; (**J**,**K**) promoter activity of croaker ATGL in HEK 293T cells after FXR or PPARα overexpression. Data are shown as means ± SEMs (*n* = 3) and were analyzed using Duncan’s multiple range test and Student’s *t*-test. The letters a–d indicate that there is a significant difference between means, * *p* < 0.05.

After finding that FXR activation could decrease the lipid deposition in the liver, we further investigated the regulation of FXR on the lipid metabolism-related genes. In cultured LYCL cells, FA treatment decreased the mRNA expression of *pparα* and *atgl* and upregulated mRNA levels of *cpt1* (*p* < 0.05) (Figure 4A,C). FXR overexpression significantly upregulated the mRNA and protein level of PPARα and CPT1, and the mRNA level of *atgl* (*p* < 0.05) (Figure 4A,B). Meanwhile, Transfection of *fxr*-siRNA significantly reduced *fxr* expression and downregulated the mRNA level of *pparα*, *cpt1* and *atgl*, and the protein level of CPT1 (*p* < 0.05) (Figure 4C,D). In primary hepatocytes, expression of *pparα*, *cpt1*, and *atgl* were significantly upregulated by GW4064 or CDCA treatment, while *fxr* knock-down decreased the expression of *pparα* and *atgl* (*p* < 0.05) (Figure 4E–G). To investigate the mechanisms underlying the above effects, we further detected the influence of FXR on the activity of croaker PPARα and ATGL promoters. The results showed that overexpression or ligands-meditated activation of FXR significantly increased the promoter activity of croaker PPARα (*p* < 0.05) (Figure 4H). Chip assays showed that the PRARα promoter fragment (−1403, −1047) coprecipitated with FXR-FLAG, and binding sites were predicted in this fragment (Figure 4I). However, the promoter activity of ATGL was not affected by FXR transfection, whereas it was significantly enhanced by PPAR overexpression. (Figure 4J,K).

### 3.5. Regulation of FXR on Expression of srebp1 and Other Lipogenesis Related Genes

**Figure 5:**
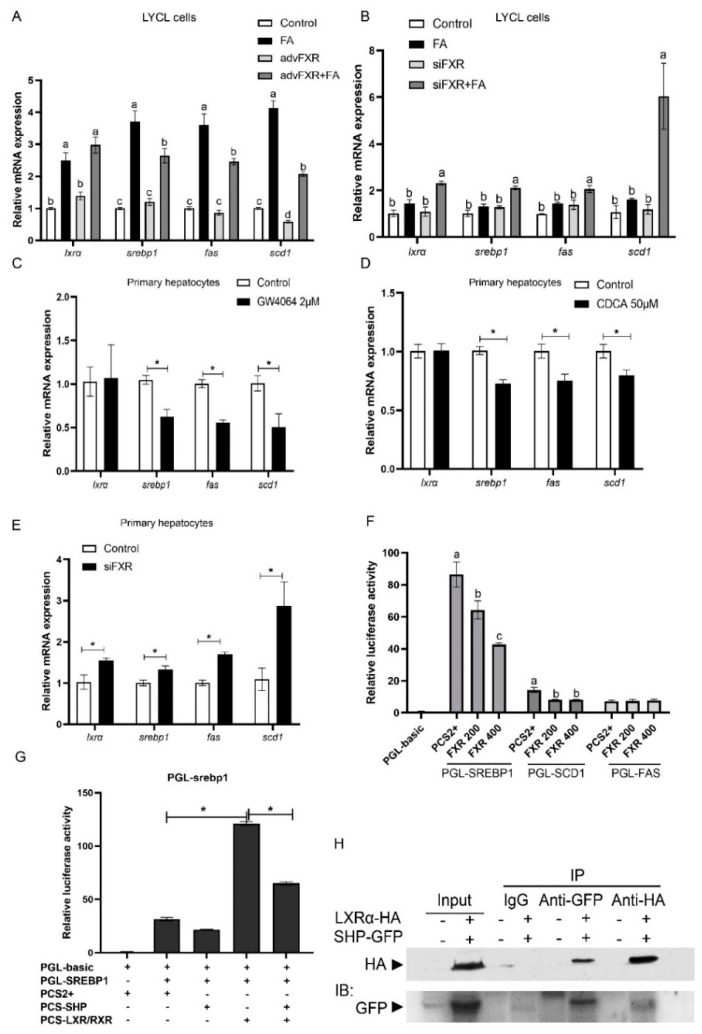
Effects of FXR on genes involved in lipogenesis of large yellow croakers: (**A**,**B**) Gene expression of *lxrα*, *srebp1*, *fas*, *and scd1* in LYCL cells after FXR overexpression or knockdown and treatment with FA; (**C**–**E**) gene expression of *lxrα*, *srebp1*, *fas*, *and scd1* in primary hepatocytes after GW4064 or CDFA treatment or FXR knockdown; (**F**) promoter activity of croaker SREBP1, SCD1, and FAS in HEK 293T cells after FXR overexpression; (**G**) promoter activity of croaker SREBP1 after LXRα and SHP overexpression; (**H**) analysis of protein interactions between LXRα and SHP of large yellow croaker. Data are shown as means ± SEMs (*n* = 3) and were analyzed using Duncan’s multiple range test and Student’s *t*-test. The letters a–d indicate that there is a significant difference between means, * *p* < 0.05.

After treatment with FA, the expression of lipogenesis genes (*lxrα*, *srebp1*, *fas*, and *scd1*) in LYCL cells was significantly higher than that in the control group (*p* < 0.05) (Figure 5A). FXR overexpression inhibited FA-induced expression of *srebp1*, *fas*, and *scd1* (*p* < 0.05) (Figure 5A). The siRNA-mediated knock-down of FXR significantly enhanced expression of *lxrα*, *srebp1*, *fas*, and *scd1* induced by FA treatment in LYCL cells (*p* < 0.05) (Figure 5B). Similarly, GW4064 and CDCA treatment downregulated the mRNA levels of *srebp1*, *fas* and *scd1* in primary hepatocytes, but FXR knock-down increased the expression of these genes (*p* < 0.05) (Figure 5C–E). Then, we further investigated the effect of FXR on the activity of SREBP1, SCD1 and FAS promoters. The results showed that FXR overexpression significantly decreased the activity of croaker SREBP1 and SCD1 promoters (*p* < 0.05) (Figure 5F). Meanwhile, the activity of croaker SREBP1 promoter was significantly increased in HEK 293T cells transfected with LXRα. The LXRα agonist significantly increased expression of *srebp1* and enhanced the inducing effect of LXRα on the promoter activity of croaker SREBP1 (Supplementary Figure S2). The promoter activity of SREBP1 was significantly lower in cells co-transfected with SHP and LXRα expression plasmids than in cells transfected with LXRα plasmid alone (*p* < 0.05) (Figure 5G). The co-IP results showed that SHP could bind to LXR through protein-protein interactions (Figure 5H).

### 3.6. HFD Reduces FXR Expression in Association with ER Stress

**Figure 6:**
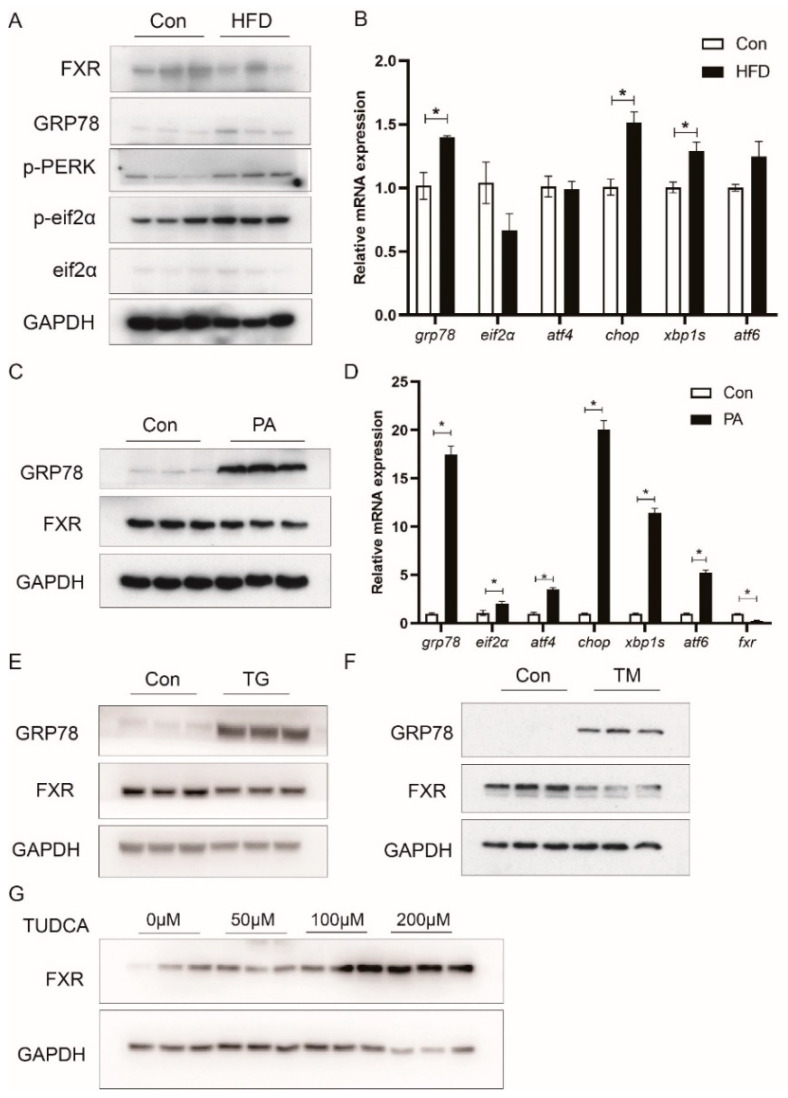
Effects of HFD and ER stress on the expression of FXR in large yellow croaker: (**A**,**B**) Immunoblots or qRT-PCR assays for FXR and ER stress markers in the liver of large yellow croakers fed HFD diet; (**C**–**F**) immunoblots or qRT-PCR assays for FXR and ER stress markers in LYCL cells after PA, TG, or TM treatment; (**G**) immunoblots for FXR in LYCL cells after TUDCA treatment. Data are shown as means ± SEMs (*n* = 3) and were analyzed using Student’s *t*-test, * *p* < 0.05.

Consistent with the above result that HFD decreased the mRNA expression of *fxr*, western blot analysis indicated that HFD decreased the protein level of FXR in the liver (Figure 6A). In addition, high-fat diets significantly increased protein levels of ER stress markers, such as GRP78, p-PERK, and p-EIF2α (Figure 6A), as well as mRNA expression of *grp78*, *chop*, and *xbp1s* (*p* < 0.05) (Figure 6B). In the LYCL cells, PA treatment enhanced the protein levels of GRP78 and mRNA expression of *grp78*, *eif2α*, *atf4*, *chop*, *xbp1s* and *atf6* while decreasing the expression of FXR (*p* < 0.05) (Figure 6C,D). Then, to explore the effect of ER stress on FXR expression, the cells were treated with ER-stress inducer (TG or TM) or inhibitor (TUDCA). TG or TM treatment markedly increased the protein level of GRP78 and decreased the expression of FXR (Figure 6E,F). Meanwhile, the FXR protein levels were significantly increased in the TUDCA group (Figure 6G).

### 3.7. ER Stress Reduces FXR Expression through the MAPK Pathway

**Figure 7:**
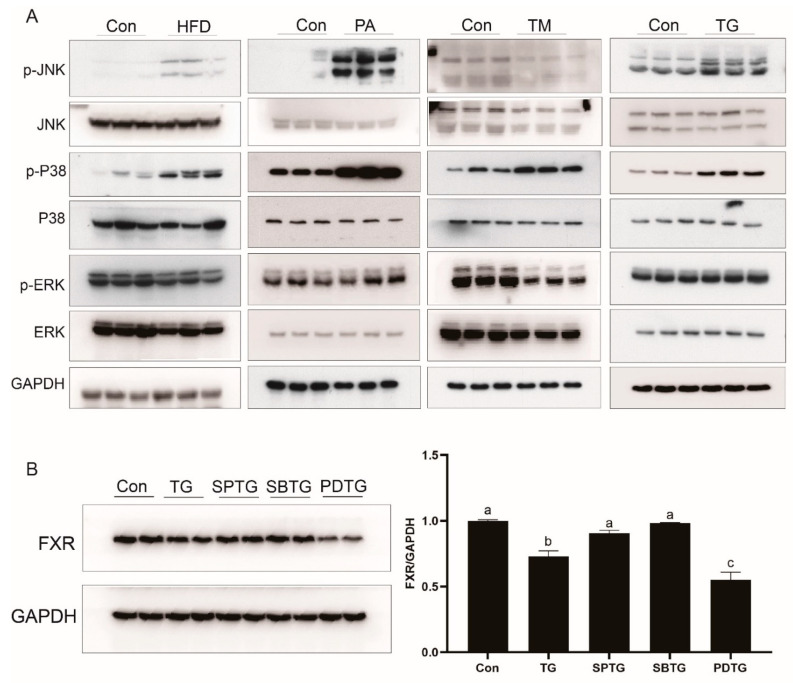
Effects of MAPK signaling pathway on the expression of FXR: (**A**) Immunoblots for MAPK signaling pathway in liver of large yellow croakers fed HFD diet and LYCL cells after PA, TM, or TG treatment; (**B**) immunoblots for FXR in LYCL cells after TG treatment alone or with MAPK inhibitors. Data are shown as means ± SEMs (*n* = 3) and were analyzed using Duncan’s multiple range test and Student’s *t*-test. The letters a–c indicate that there is a significant difference between means, *p* < 0.05.

As compared with the control group, high-fat diets increased the phosphorylation levels of JNK and P38. Similarly, in the LYCL cells, the PA or TG treatment increased the phosphorylation levels of JNK and P38. However, the TM treatment only increased the phosphorylation level of P38 while decreasing the phosphorylation levels of JNK and ERK (Figure 7A). To explore the involvement of the MAPK pathway in the regulation of FXR, cells were treated with TG and MAPK inhibitors. As compared with the TG group, SP600125 (JNK inhibitor) and SB23580 (P38 inhibitors) treatment significantly increased FXR protein levels while PD98059 (ERK inhibitors) treatment decreased it (Figure 7B).

### 3.8. HNF1α Is Involved in Effects of ER Stress and MAPK Pathway on FXR

**Figure 8:**
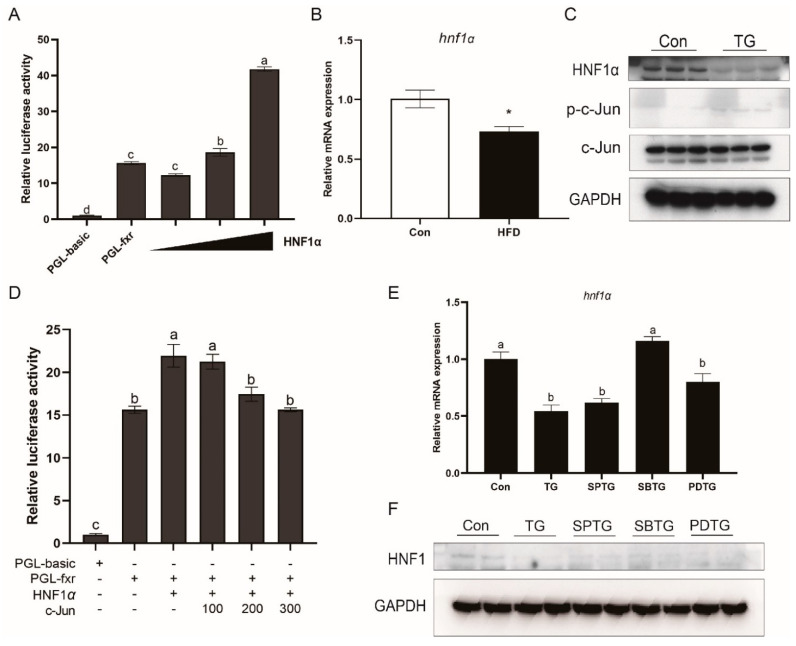
HNF1α is involved in er stress effect on FXR. (**A**) Promoter activity of croaker FXR in HEK 293T cells after HNF1α overexpression. (**B**) qRT-PCR assays for HNF1α in the liver of large yellow croakers fed HFD diet. (**C**) Immunoblots for HNF1α and c-Jun in LYCL cells after TG treatment. (**D**) Promoter activity of croaker FXR in HEK 293T cells after HNF1 overexpression alone or co-transfected with c-Jun. (**E**,**F**) Immunoblots or qRT-PCR assays for HNF1α in LYCL cells after TG treatment alone or with MAPK inhibitors. Data are shown as means ± SEMs (*n* = 3) and were analyzed using Duncan’s multiple range test and Student’s *t*-test. The letters a–c indicate that there is a significant differences between the means, * *p* < 0.05.

HNF1α was also shown to be an FXR regulator in large yellow croaker. The promoter activity of croaker FXR was significantly increased by HNF1α overexpression (*p* < 0.05) (Figure 8A). In addition, *hnf1α* expression was significantly lower in the HFD group as compared with that in the control group (*p* < 0.05) (Figure 8B). In the LYCL cells, TG treatment decreased the HNF1α protein level and increased the phosphorylation levels of c-Jun (Figure 8C). The luciferase reporter assay showed that co-expression of c-Jun with HNF1α significantly suppressed the effect of HNF1α on the FXR promoter (*p* < 0.05) (Figure 8D). Furthermore, as compared with the cells treated with TG alone, the mRNA expression and protein level of HNF1α were significantly higher in cells after SB23580 treatment (*p* < 0.05) (Figure 8E,F).

## 4. Discussion

In the present study, high-fat diets induced lipid deposition in the liver of large yellow croakers and caused liver damage, which is also the case in other fish fed high-fat diets [32,33,34,35]. However, fatty liver in mammals is also a major health problem that needs to be resolved. FXR is studied as a target gene to treat non-alcoholic fatty liver disease [11,36]. The FXR agonist treatment has been shown to have the effects of reducing triglycerides in the liver of mice and rat models [5,8,37,38]. In our previous study, CDCA and GW4064 were confirmed to be effective agonists of FXR in large yellow croakers [28]. In the present study, the in vivo and in vitro results showed that CDCA- and GW4064-mediated activation of FXR decreased lipid deposition in liver and primary hepatocytes. Moreover, overexpression of FXR could decrease the triglyceride content in the LYCL cells. These results suggest that FXR is involved in fish liver lipid metabolism regulation and may be used as an effective target for decreasing liver lipid deposition in large yellow croakers.

Furthermore, we explored the molecular mechanism through which FXR influenced lipid metabolism. The results showed that feeding an HFD and FA treatment decreased expression of lipid catabolism related genes (*pparα* and *atgl)* and increased expression of lipogenesis-related genes (*srebp1*, *fas*, and *scd1*). Overexpression and agonists-mediated-activation of FXR increased protein and mRNA expression of PPARα, CPT1, and *atgl*, as well as decreased mRNA expression of *srebp1*, *fas*, and *scd1*, while knocking down FXR decreased expression of lipid catabolism-related genes, and increased lipogenesis-related genes expression. These findings were consistent with previous findings in mammals [3,5,39,40], which indicated that the reducing effect of FXR on liver lipid deposition may result from an increase in lipolysis and fatty acid oxidation, and a decrease in lipogenesis. PPARα has been recognized as a key regulator of fatty acid β-oxidation in mammals [41] and fish [42]. In the present study, croaker FXR affected the expression of PPARα, and FXR could induce the promoter activity of PPARα through binding to the promoter of PPARα. These results indicate that the expression of PPARα is regulated by FXR in large yellow croakers. Studies in human have also shown that FXR regulates the expression of PPARα [9,40]. However, in mice, the activity of the PPARα promoter was not influenced by FXR [40]. These findings demonstrated that the function of FXR may have diversity in different species. In addition, the present results showed that FXR induced the expression of *atgl* which is an important lipase that regulates triglycerides turnover and directs released fatty acids to oxidative pathways [43]. Correia et al. [9] also showed that FXR activation increased the expression of *atgl*. However, we did not find an effect of FXR on the activity of croaker ATGL promoter, while PPARα overexpression could significantly increase the promoter activity of ATGL. These findings suggest that croaker FXR may increase mRNA expression of *atgl* by PPARAα. In addition to increasing of lipolysis and oxidation, FXR has also been shown to suppress *de novo* lipogenesis [4]. In mammals, FXR activation decreases expression of *srebp-1*c and its target genes such as *fas* and *scd1*, and SHP is essential for the suppression effect of FXR on *srebp-1c* [5]. Consistently with this, the findings of this investigation also showed that FXR activation decreased the expression of *srebp1*, *fas* and *scd1*. Overexpression of croaker FXR decreased the activity of SREBP1 and SCD1 promoter, and SHP could decrease the LXRα-induced activity of SREBP1 promoter by directly binding to LXRα in fish. In conclusion, these findings suggest that FXR activation can prevent hepatic lipid accumulation by promoting expression of lipid catabolism-related genes and suppressing expression of lipogenesis-related genes.

After understanding the role of FXR in regulating lipid metabolism, we further investigated how FXR expression was regulated in fish fed high-fat diets. In the present studies, the protein and mRNA level of FXR was decreased in the liver of fish fed high-fat diets and hepatocytes with PA treatment. In mammals, FXR is downregulated in patients with NAFLD or liver injury, and obese rodents [23,44]. Xiong et al. [24] reported that activation of ER stress resulted in a decrease in hepatic FXR expression in aging mice. Studies in teleost also showed that high-fat diets induced ER stress in liver [25,26]. Then, we investigated whether high-fat diets could affect the expression of FXR by ER stress. The results showed that feeding high-fat diets and PA treatment indeed induced ER stress in large yellow croakers. TM and TG mediated-activation of ER stress decreased expression of FXR, and inhibiting ER stress by TUDCA increased the protein level of FXR. These findings suggested that ER stress was involved in the HFD-induced decrease in FXR. The study in mice showed that ER stress down-regulates FXR expression by suppressing HNF1α transcriptional activity via the JNK/c-Jun pathway activation [24]. However, in the present study, HFD, PA, TM and TG showed different effects on the activation of the MAPK pathway. HFD, PA, and TG were shown to enhance the phosphorylation levels of JNK and p38 MAPK, whereas TM increased P38 phosphorylation while decreasing JNK and ERK phosphorylation. These findings indicated that MAPK was involved in the effect of ER stress on FXR not just through the JNK/c-Jun pathway. Therefore, we further inhibited three MAPK pathways respectively. SP600125 (JNK inhibitor) and SB23580 (P38 inhibitors) treatment prevented the inhibiting effect of ER stress on FXR, which suggested that P38 MAPK may also be a factor affecting FXR. In line with findings in mammals [24,45], we detected that HNF1α was a regulator of FXR, and co-expression of c-Jun suppressed the transcriptional activity of HNF1α on FXR promoter in large yellow croaker. Moreover, HFD and TG treatment decreased expression of HNF1α in large yellow croakers while P38 inhibition could relieve TG-decreased expression of HNF1α. Together, these findings suggest that high-fat diets may reduce transcriptional activity and expression of HNF1α via ER-stress-mediated-activation of JNK and P38 MAPK, and thereby inhibit FXR expression in large yellow croakers.

In conclusion, FXR is a key lipid metabolism regulator in the liver of teleost. Activation of FXR can prevent HFD-induced abnormal lipid accumulation in liver of large yellow croakers, mainly through increasing expression of lipolysis and β-oxidation-related genes and repressing expression of lipogenesis-related genes. This study also confirms that high-fat diets can suppress FXR expression by ER-stress-mediated-activation of the JNK and P38 MAPK pathways.

## Data Availability

Not applicable.

## Supplementary Materials

The following are available online at https://www.mdpi.com/article/10.3390/nu13124343/s1, Table S1. Formulation and proximate analysis of the experimental diets^1^; Table S2. Primers used for qPCR analysis ^1^; Table S3. Primers used for plasmid construction ^1^; Figure S1. Effects of HFD and supplementation of CDCA on the survival (A) and weight gain rate of large yellow croakers. Data are shown as means ± SEMs (*n* = 3) and were analyzed using Duncan’s multiple range test. Labeled means without a common letter differ, *p* < 0.05; Figure S2. Effects of LXRα on the expression of srebp1 and other lipogenesis related genes. (A) Effects of GW3965 (LXRα agonist) treatment on expression of srebp1, fas and scd1 in cells of large yellow croaker. (B) Effects of GW3965 and croaker LXRα overexpression on the promoter activity of SREBP1 in HEK 293T. Data are shown as means ± SEMs (*n =* 3) and were analyzed using Student’s *t*-test, * *p* < 0.05.

## Abbreviations

ALT, alanine aminotransferase; AST, aspartate aminotransferase; ATF4, activating transcription factor 4; ATF6, activating transcription factor 6; ATGL, adipose triglyceride lipase; APOB100, Apolipoprotein B100; BSEP, bile salt export pump; CDCA, chenodeoxycholic acid; CPT1, carnitine palmitoyltransferase I; CHOP, C/EBP homologous protein; FXR, farnesoid X receptor; FAS, fatty acid synthase; EIF2α, eukaryotic initiation factor 2α; GAPDH, glyceraldehyde-3-phosphate dehydrogenase; GRP78, glucose-regulated protein 78; GFP, Green fluorescent protein; HFD, high-fat diet; HNF1α, hepatocyte nuclear factor 1α; HFD-CDCA 300, HFD + 300 mg/kg CDCA; HFD-CDCA 900, HFD + 900 mg/kg CDCA; LXRα, liver X receptor α; LDLR, low density lipoprotein receptor; MTP, microsomal triglyceride transfer protein; NEFA, non-esterified fatty acid; PPARα, proliferator-activated receptor α; PA, palmitic acid; SHP, small heterodimer partner; SREBP1, sterol regulatory element binding protein 1; SCD1, stearoyl-CoA desaturase 1; TG, thapsigargin; TM, tunicamycin; TUDCA, tauroursodeoxycholate; XBP1s, X-box-binding protein 1; SPTG, thapsigargin and SP600125; SBTG, thapsigargin and SB3580; PDTG, thapsigargin and PD98059.
