# Supplementary material for: FXR, a Key Regulator of Lipid Metabolism, Is Inhibited by ER Stress-Mediated Activation of JNK and p38 MAPK in Large Yellow Croakers (Larimichthys crocea) Fed High Fat Diets
Source: Nutrients. 2021 Dec 1;13(12):4343. doi: 10.3390/nu13124343 (PMC8706856; doi:10.3390/nu13124343)
Supplement: Supplementary file 1 [file nutrients-13-04343-s001.zip › nutrients-1474985-supplementary.pdf]

**Table S1.** Formulation and proximate analysis of the experimental diets [1].

| | Diets | | | |
|---|---|---|---|---|
| **Ingredients g/kg** | **Control** | **HFD** | **HFD-CDCA 300** | **HFD-CDCA 900** |
| Fish meal | 390 | 390 | 390 | 390 |
| Soybean meal | 200 | 200 | 200 | 200 |
| Wheat meal | 230 | 230 | 230 | 230 |
| Wheat starch | 60 | 60 | 60 | 60 |
| Fish oil | 60 | 120 | 120 | 120 |
| Soybean lecithin | 15 | 15 | 15 | 15 |
| Vitamin premix[2] | 20 | 20 | 20 | 20 |
| Mineral premix[3] | 20 | 20 | 20 | 20 |
| Attractant[4] | 3 | 3 | 3 | 3 |
| Mold inhibitor[5] | 1 | 1 | 1 | 1 |
| CDCA | | | 0.3 | 0.9 |
| Proximate composition (% dry matter) | | | | |
| Crude protein | 45.2 | 45.5 | 45.1 | 45.4 |
| Crude lipid | 11.5 | 17.9 | 17.1 | 17.7 |

[1] CDCA, chenodeoxycholic acid; HFD, high-fat diet; HFD-CDCA300, high-fat diet supplemented with 300 mg/kg CDCA; HFD-CDCA 900, high-fat diet supplemented with 900 mg/kg CDCA. [2]Vitamin premix (mg or g/kg diet): cholecalciferol, 5 mg; retinol acetate, 32 mg; thiamin 25 mg; cyanocobalamin, 10 mg; riboflavin, 45 mg; pyridoxine HCl, 20 mg; ascorbic acid, 2000 mg; alpha-tocopherol (50%), 240 mg; menadione, 10 mg; pantothenic acid, 60 mg; inositol, 800 mg; niacin acid, 200 mg; folic acid, 20 mg; biotin (2%), 60 mg; choline chloride (50%), 4000 mg; microcrystalline cellulose, 12.47 g. [3]Mineral premix (mg or g/kg diet): $CuSO_4{\cdot}5H_2O$, 10 mg; Ca $(IO_3)_2{\cdot}6H_2O$ (1%), 60 mg; $CoCl_2{\cdot}6H_2O$ (1%), 50 mg; $FeSO_4{\cdot}H_2O$, 80 mg; $MgSO_4{\cdot}7H_2O$, 1200 mg; $MnSO_4{\cdot}H_2O$, 45 mg; $NaSeSO_3{\cdot}5H_2O$ (1%), 20 mg; $ZnSO_4{\cdot}H_2O$, 50 mg; $CaH_2PO_4{\cdot}H_2O$, 10 g; Zeolite, 8.485 g. [4]Attractants: glycine and betaine [5]Mold inhibitor: contained 50% calcium propionic acid and 50% fumaric acid.

**Table S2.** Primers used for qPCR analysis [1.]

| Genes | Forward Sequences (5′–3′) | Reverse Sequences (5′–3′) | Accession No. |
|---|---|---|---|
| *atgl* | CCATGCATCCGTCCTTCAACC | GAGATCCCTAACCGCCCACT | GQ372967.1 |
| *apob100* | AGAGTGTTGTCCAGGATAAA GATGC | CAGGGCTCAGGGTCTCAGTC | XM_027284282.1 |
| *atf4* | GCCGTTATTCTGCTCCATCTT CT | AGACCTTACCCTGAGCCCACAT | XM_019253810.2 |
| *atf6* | CAGATAATAAGGAGGCTGAG AGTGC | CGTAGGTATGATGAGGTGCGTA GT | XM_010730227.3 |
| *bsep* | TGATGGCGGCGGGTAGTTT | GCAGCAGTGTAGTTCCTCTTCC | XM_027291804.1 |
| *cd36* | GAGCATGATGGAAAATGGTT CAAAG | GAGCATGATGGAAAATGGTTCA AAG | KM593122.1 |
| *chop* | TCTGGATGTTCTGGAGAGTTG TTC | AGGATGATGATGAGGTGTGATG C | XM_010732112.3 |
| *cpt1* | TGGGTGCTGGTGGCGGATTT | GGGCTGGAGAACTTGCTGGAGA | XM_019255573.2 |
| *eif2α* | ATGCCGGGGCTCAGCTGTC | CTAGTCCTCTGCTTTGGCCTCC | XM_019277014.2 |
| *fas* | ACTCCTATGTGGCAGCATAGA C | GTTTCAGCCTCAGACTCTTTGCC | KP889061.1 |
| *fxr* | TGGAGGAAAGGATACGCAAG AGTG | TGTCAGGATGGTTACGGTGGTG | XM_010737684 |

| | | | |
|---|---|---|---|
| *gapdh* | GACAACGAGTTCGGATACAGC | CAGTTGATTGGCTTGTTTGG | XM_010743420.3 |
| *grp78* | GGTGGCGATGACAAGCAAAC | CTGAGAACAGCAGCAACAAGC | XM_010738795.3 |
| *lxrα* | TCTCAAAAGGAATGAACGAC | TAAGGTTGCTGTAGCCTCTCA | XM_019273432.2 |
| *ldlr* | ACATAAGCGCCGGTGCTGTT | TACGATGTCCTCTGGCTGATTC | KM593127.1 |
| *mtp* | ATGTCCAAAATGTTCTCCATGTCTG | ATGTCAATAGCCAACCCTCCTTG | KP027412.1 |
| *pparα* | GTCAAGCAGATCCACGAAGCC | TGGTCTTTCCAGTGAGTATGAGCC | XM_027291871.1 |
| *scd1* | ACAAGCTCTCCGTGGTGGTCAT | GCCAGGTAGCGTTCAGCAACAT | KP202157.1 |
| *shp* | GCGACGGACAGTGTGCTTGAA | ACTGGTCGTTTGGTGGCATCTG | KY745777.1 |
| *srebp1* | CCAAGACAGAGGAGTGCGAGAC | TCATTTGCTGGCAGTCGTGGAG | KP342262.1 |
| *xbp1s* | GTCTTCTGAGTCCGCAGCAGGTG | AGGATGTCCAGAATGCCCAGTAG | XM_019264508.2 |
| *β-actin* | CTACGAGGGTTATGCCCTGCC | TGAAGGAGTAACCGCGCTCTG | GQ168793.1 |

[1] *atgl*, adipose triglyceride lipase; *apob100,* Apolipoprotein B100*; atf4,* activating transcription factor 4*; atf6*, activating transcription factor 6*; bsep,* bile salt export pump*; chop,* C/EBP homologous protein*; cpt1,* carnitine palmitoyltransferase I*; eif2α*, eukaryotic initiation factor 2α*; fas*, fatty acid synthase*; fxr*, farnesoid X receptor; *gapdh*, glyceraldehyde-3-phosphate

dehydrogenase; *grp78,* glucose-regulated protein 78*; lxrα,* liver X receptor α*; ldlr*, low density lipoprotein receptor*; mtp,* microsomal triglyceride transfer protein*; pparα,* proliferator-activated receptor α*; scd1*, stearoyl-CoA desaturase 1; *shp*, small heterodimer partner; *srebp1,* sterol regulatory element binding protein 1*; xbp1s*, X-box-binding protein 1.

**Table S3.** Primers used for plasmid construction [1].

| Primers | Forward Sequences (5′–3′) | Reverse Sequences (5′–3′) |
|---|---|---|
| pGL-SREBP1 | GGGGTACCAGGTTAAGTATGGTTGCCAGGTAA | CCGCTCGAGCTTTACTCCTGTGTGCGG |
| pGL-PPARα | CCCTCGAGGGCAGCAGCAGCAATCGTCATCGT | CCAAGCTTTGTCTGTCAGCGGAGTCAGGTGTTCT |
| pGL-ATGL | GGGGTACCGCCTCTGCTCTGCTTACTTCCTTGTG | CCGCTCGAGCGAAGGAGATGTTCCACGGCGAGTCT |

[1] sterol regulatory element binding protein 1; PPARα, proliferator-activated receptor α; ATGL, adipose triglyceride lipase.

A

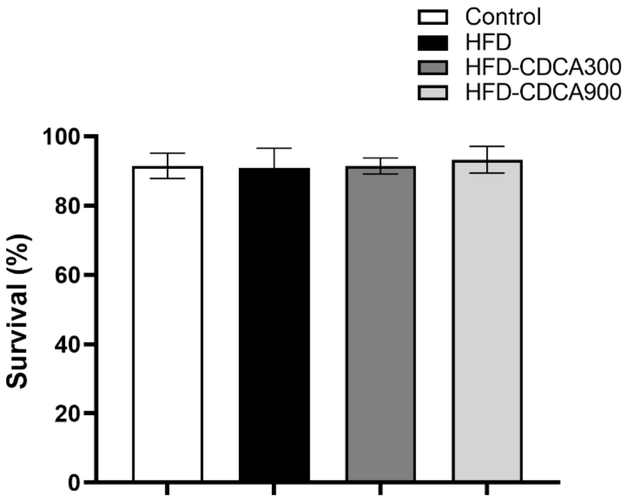


B

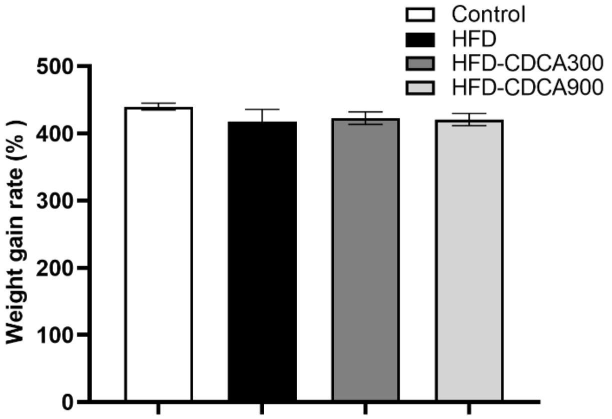


**Figure S1.** Effects of HFD and supplementation of CDCA on the survival (**A**) and weight gain rate (**B**) of large yellow croakers. Data are shown as means ± SEMs ($n$ = 3) and were analyzed using Duncan's multiple range test. Labeled means without a common letter differ, $p < 0.05$.

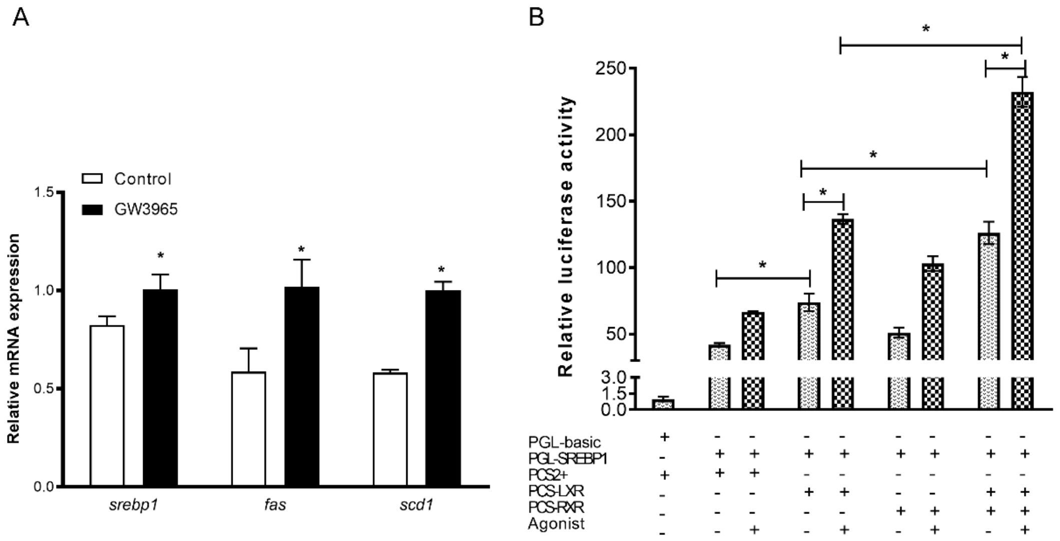


**Figure S2.** Effects of LXRα on the expression of *srebp1* and other lipogenesis related genes. (**A**) Effects of GW3965 (LXRα agonist) treatment on expression of srebp1, fas and scd1 in cells of large yellow croaker. (**B**) Effects of GW3965 and croaker LXRα overexpression on the promoter activity of SREBP1 in HEK 293T. Data are shown as means ± SEMs ($n$ = 3) and were analyzed using Student’s t-test, * $p < 0.05$.
